# Supplementary material for: Charge injection engineering at organic/inorganic heterointerfaces for high-efficiency and fast-response perovskite light-emitting diodes
Source: Nat Commun. 2023 Oct 13;14:6441. doi: 10.1038/s41467-023-41929-9 (PMC10575909; doi:10.1038/s41467-023-41929-9)
Supplement: Supplementary file 1 — Supplementary Information [file 41467_2023_41929_MOESM1_ESM.pdf]

Supplementary Information for:

Charge Injection Engineering at Organic/Inorganic Heterointerfaces for High-Efficiency and Fast-Response Perovskite Light-Emitting Diodes

Zhenchao Li[1,2,†], Ziming Chen[1,3,†,*], Zhangsheng Shi[4], Guangruixing Zou[5], Linghao Chu[1], Xian-Kai Chen[4,5,6,7,8,*], Chujun Zhang[9], Shu Kong So[9], Hin-Lap Yip[1,5,10,11,*]

[1] State Key Laboratory of Luminescent Materials and Devices, Institute of Polymer Optoelectronic Materials and Devices, School of Materials Science and Engineering, South China University of Technology, 381 Wushan Road, Guangzhou, 510640, P. R. China.

[2] State Key Laboratory of Advanced Materials and Electronic Components, Guangdong Fenghua Advanced Technology Holding Co. Ltd., Zhaoqing, Guangdong 526020, China.

[3] Department of Chemistry and Centre for Processible Electronics, Imperial College London, London W12 0BZ, United Kingdom.

[4] Department of Chemistry, City University of Hong Kong, Tat Chee Avenue, Kowloon, Hong Kong.

[5] Department of Materials Science and Engineering, City University of Hong Kong, Tat Chee Avenue, Kowloon, Hong Kong.

[6] Hong Kong Institute for Advanced Study, City University of Hong Kong, Tat Chee Avenue, Kowloon, Hong Kong.

[7] Institute of Functional Nano & Soft Materials (FUNSOM), Soochow University, Suzhou, 215123, Jiangsu, PR China.

[8] Jiangsu Key Laboratory of Advanced Negative Carbon Technologies, Soochow University, Suzhou, 215123, Jiangsu, PR China.

[9] Department of Physics and Institute of Advanced Materials, Hong Kong Baptist University, Kowloon Tong 999077, Hong Kong SAR, P.R. China.

[10] School of Energy and Environment, City University of Hong Kong, Tat Chee Avenue, Kowloon, Hong Kong.

[11] Hong Kong Institute for Clean Energy, City University of Hong Kong, Tat Chee Avenue, Kowloon, Hong Kong.

[†] These authors contributed equally to this work.

[*] Correspondence: z.chen@imperial.ac.uk (Z. C.), xkchen@suda.edu.cn (X.-K. C.), a.yip@cityu.edu.hk (H.-L. Y.)

This file includes:

Supplementary Figures 1–19

Supplementary Table 1

Supplementary References

Supplementary Figures

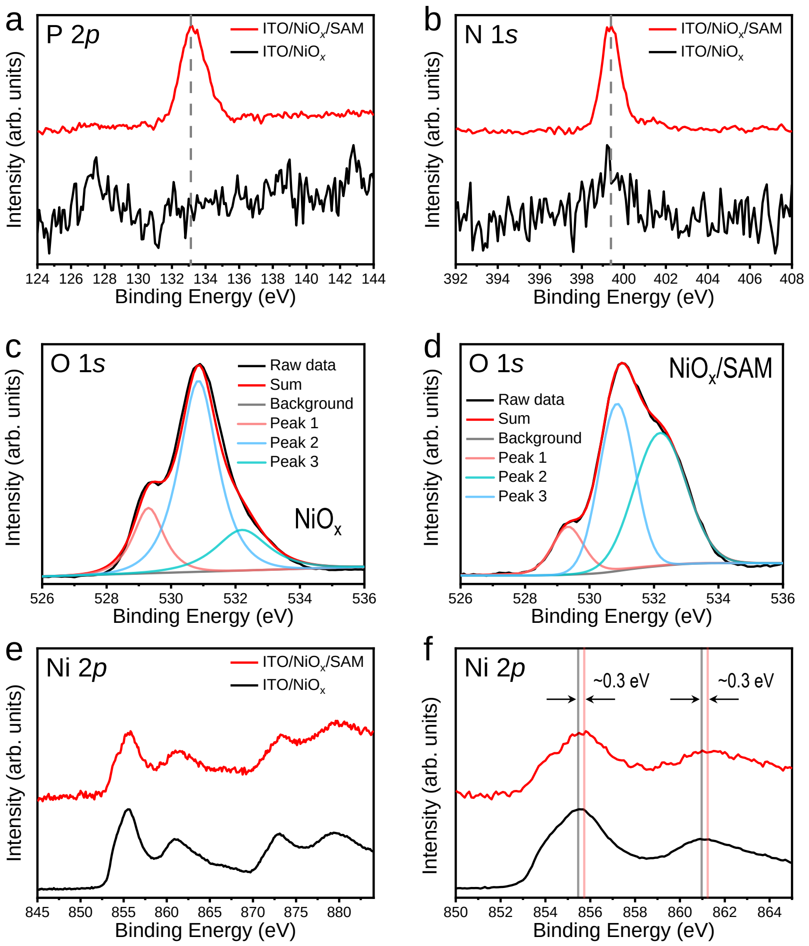

Supplementary Figure 1 | XPS analysis of the NiO$_x$ and NiO$_x$/SAM samples. a The P 2*p* signal. **b** The N 1*s* signal. **c** and **(d)** the O 1*s* signal of NiO$_x$ and NiO$_x$/SAM samples, respectively. **e** The Ni 2*p* signal and **(f)** the Zoom-in Ni 2*p* signal of NiO$_x$ and NiO$_x$/SAM samples, respectively. Compared with the ITO/NiO$_x$ sample, the P 2*p* signal at 133.2 eV and N 1*s* signal at 399.2 eV confirmed the presence of 2PACz molecules on NiO$_x$ surface. The high-resolution spectrum of the O 1*s* region elucidates the contribution of three different oxygen species. The fitted peak 1 (~529.2 eV), peak 2 (~530.8 eV), and peak 3 (~532.2 eV) are attributed to a typical Ni–O bond in NiO crystal, low-oxygen-coordinated defect site and the surface-adsorbed oxygen species, as well as interfacial interaction between Ni and O (from organic groups), respectively[1,2]. After the deposition of SAM, the relative intensity of peak 3 apparently increases, suggesting a large amount of 2PACz molecules interact with NiO$_x$ surface via the Ni–O interactions. In addition, the whole Ni 2*p* spectrum shifts towards a higher binding energy side by ~0.3 eV after the SAM treatment. The observed chemical shift suggests that the oxygen atoms in 2PACz are contributing lone pairs of electrons to the vacant orbitals of Ni atoms. As a result, coordinate covalent bonds of P=O···Ni are formed at the interface, as illustrated in Fig. 1a [3].

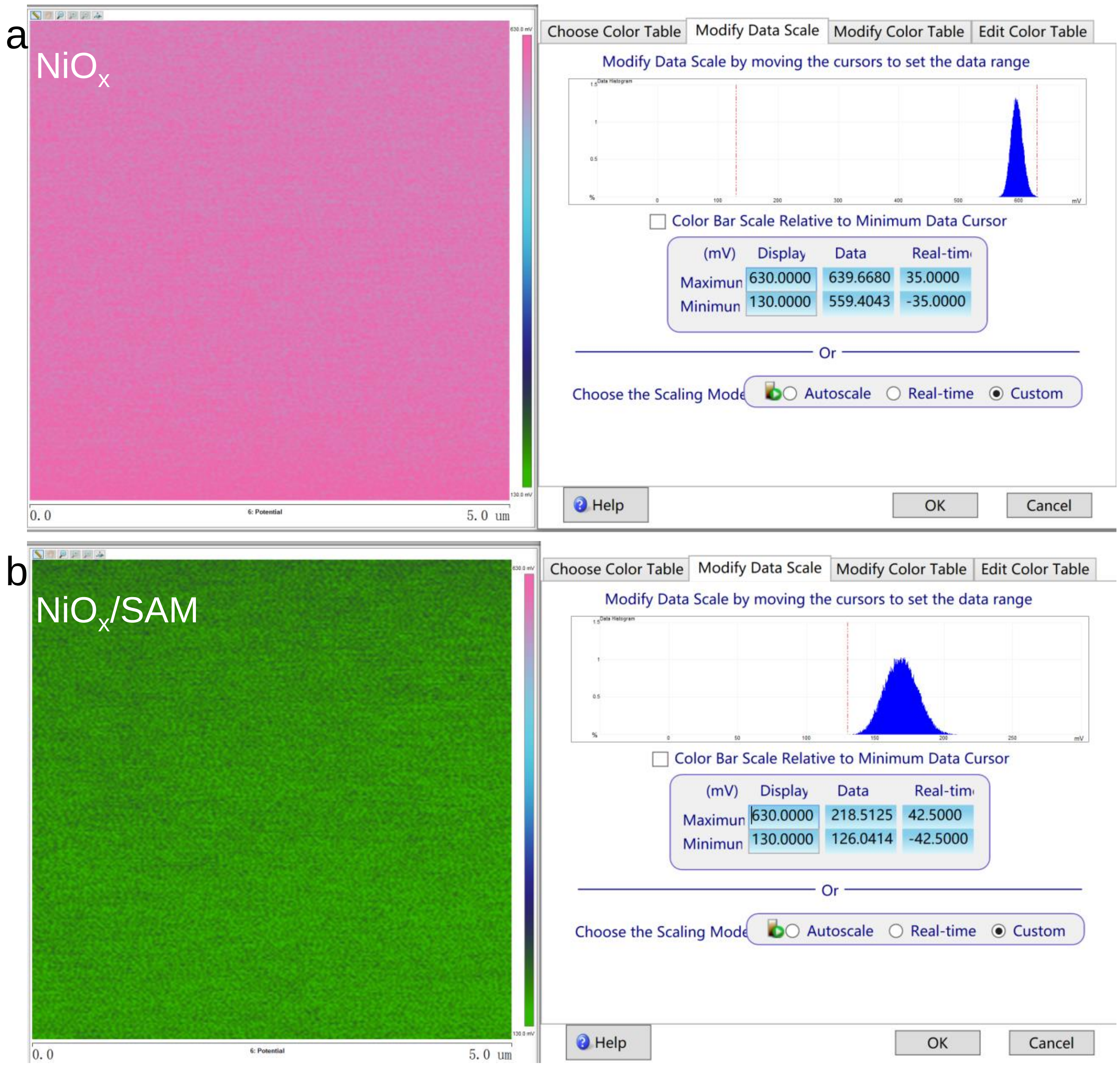

Supplementary Figure 2 | Distribution of surface potential of each pixel in Fig. 1c. a NiO_x surface and **b** NiO_x/SAM surface. In the NiO_x/SAM surface, all the pixels have surface potentials between 140–210 mV, which show in green color, and no pixel with a surface potential of 560–630 mV (in pink color) is observed. This result suggests that no NiO_x surface was exposed in the NiO_x/SAM film and SAM covers the NiO_x surface in high coverage. The results were analyzed by NanoScope Analysis 3.0 software.

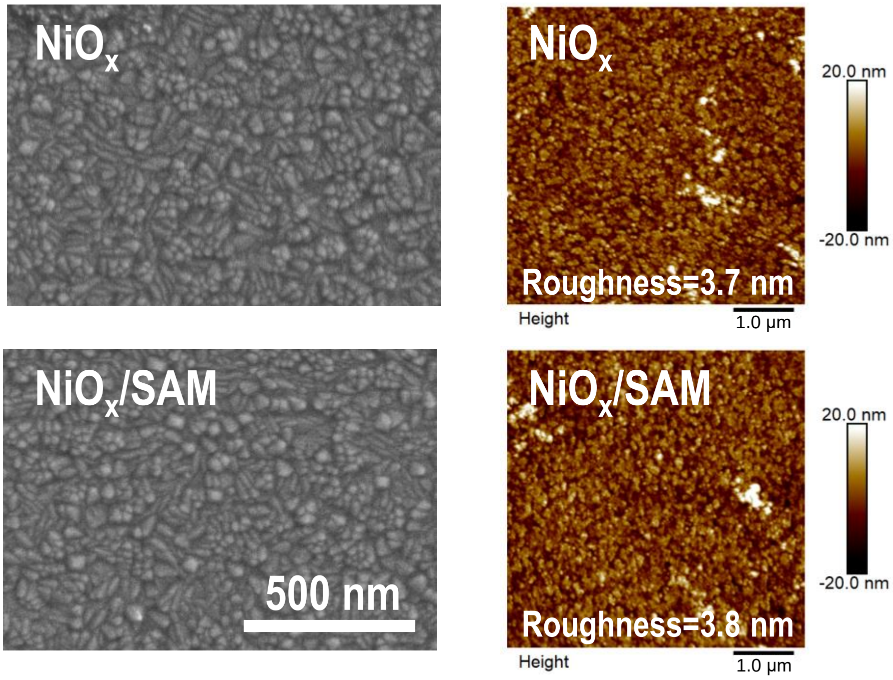

Supplementary Figure 3 | Morphologies of NiO_x and NiO_x/SAM films. Left panel: SEM images; Right panel: AFM images. No obvious NiO_x morphology and roughness changes were seen after the deposition of 2PACz.

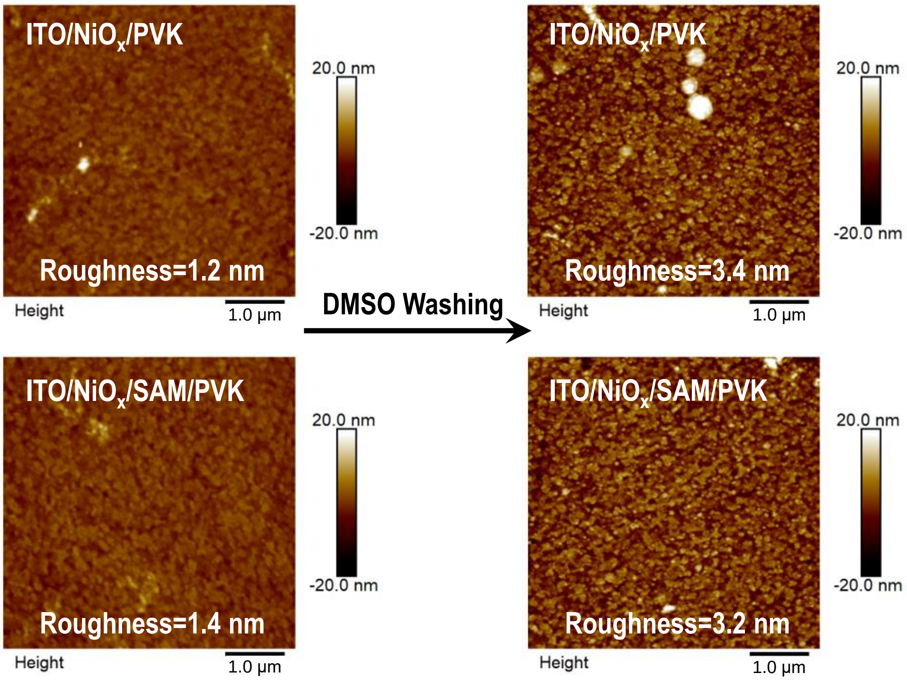

Supplementary Figure 4 | Morphology change of PVK films on NiO_x and NiO_x/SAM substrates. Left panel: before DMSO washing; Right panel: after DMSO washing. These results suggest that the morphology of the PVK layer was affected by DMSO washing.

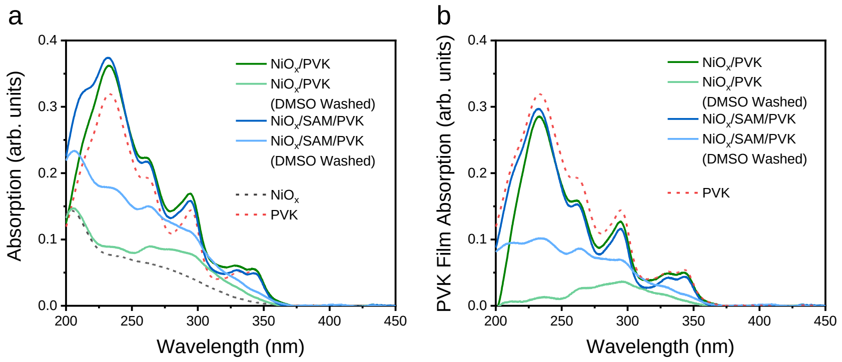

Supplementary Figure 5 | PVK layer thickness change after DMSO washing. a Absorption spectra of various films on quartz substrates. **b** Absorption spectra of PVK layers after subtracting NiO_x absorption. The PVK layer absorption dramatically decreased after DMSO washing, and the estimated PVK thicknesses in NiO_x/PVK and NiO_x/SAM/PVK decreased from ~31 nm (before washing, measured by a profilometer) to ~5.4 nm and ~17.0 nm (after washing, calculated by assuming a linear relationship between the film thickness and integral absorption intensity), respectively, suggesting that the SAM effectively strengthened the adhesion of the PVK layer. The spectrum shape of the PVK remaining in the NiO_x/SAM/PVK tri-layer was similar to that before washing, while the spectrum shape of NiO_x/PVK clearly changed before and after DMSO washing, suggesting possible changes in the morphology, film coverage and/or stacking of the PVK layer in NiO_x/PVK.

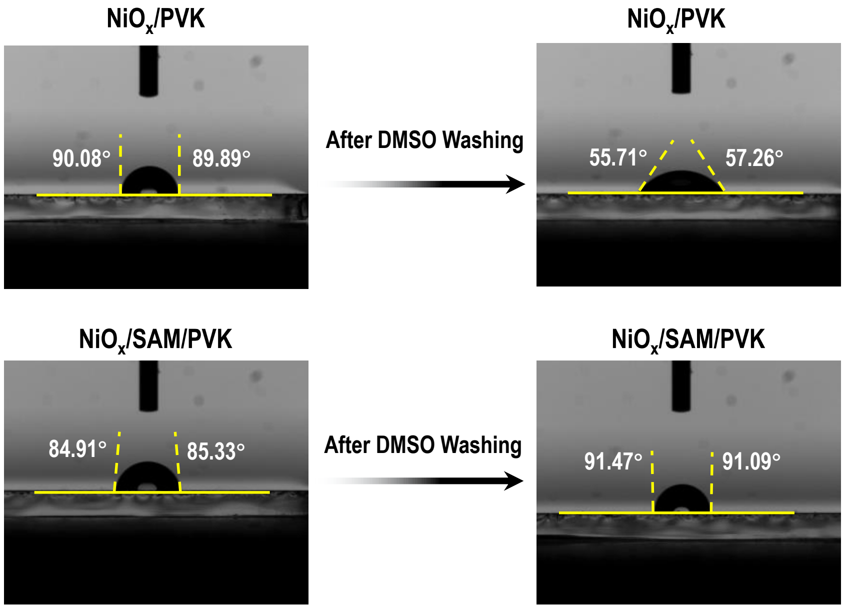

Supplementary Figure 6 | Contact angle measurements of NiO_x/PVK and NiO_x/SAM/PVK films before and after DMSO washing. The similar contact angles of the NiO_x/PVK and NiO_x/SAM/PVK samples before DMSO washing suggest similar surface nature of these two surfaces.

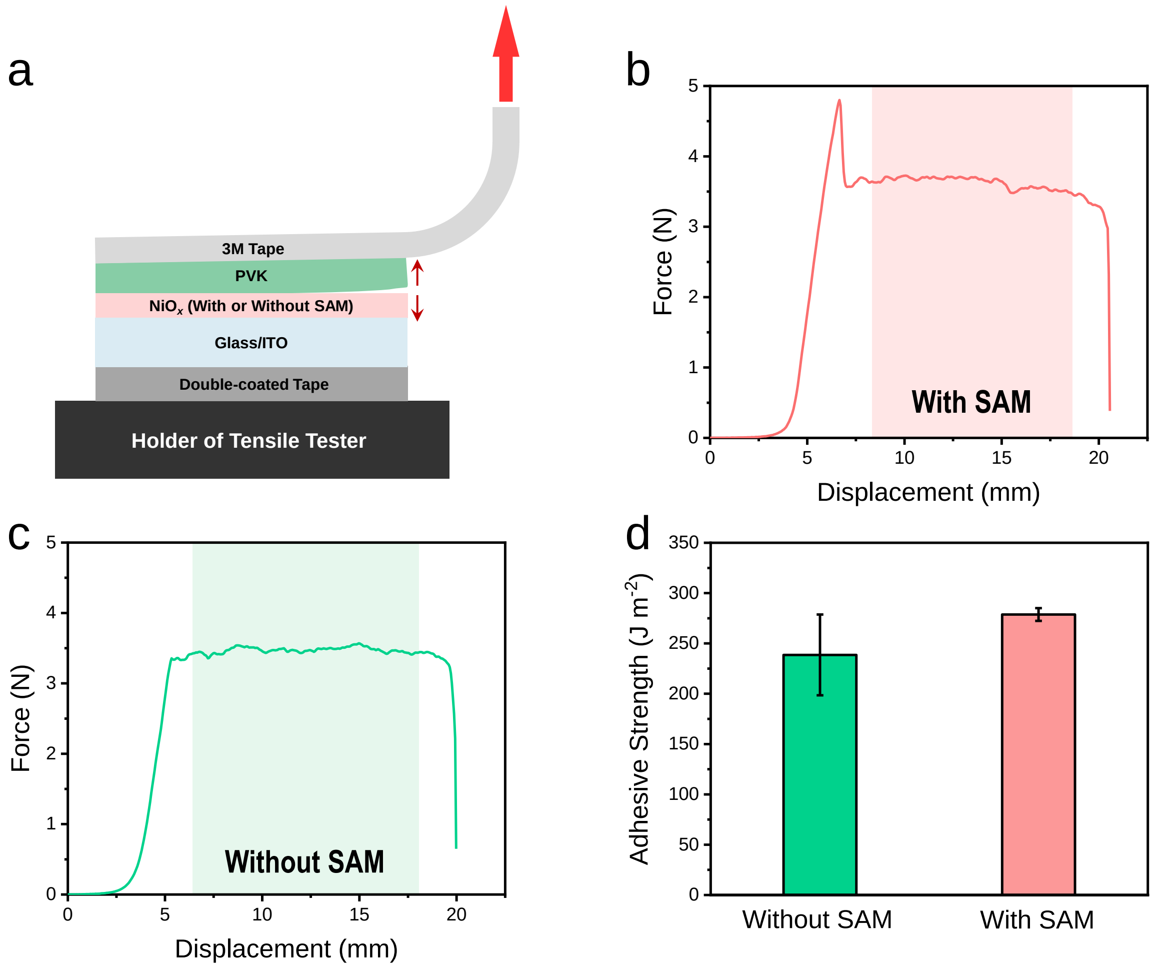

Supplementary Figure 7 | The adhesion strength measurement between the PVK/SAM and PVK/NiO$_x$ interfaces. a The schematic figure of the mechanical adhesion strength test. The force-displacement curves of (**b**) with SAM and (**c**) without SAM layer. The necessary force to peel off the PVK layer is defined as the average value of the plateau of the curve (marked in color background), which demonstrates the stable process during the layer peeling off. **d** The statistical data of the calculated adhesive strength of 10 samples (with and without SAM). The adhesion strength is calculated via dividing the necessary force by the width of tape (12.7 mm). The error bars show the highest and lowest adhesive strength values for samples without and with SAM.

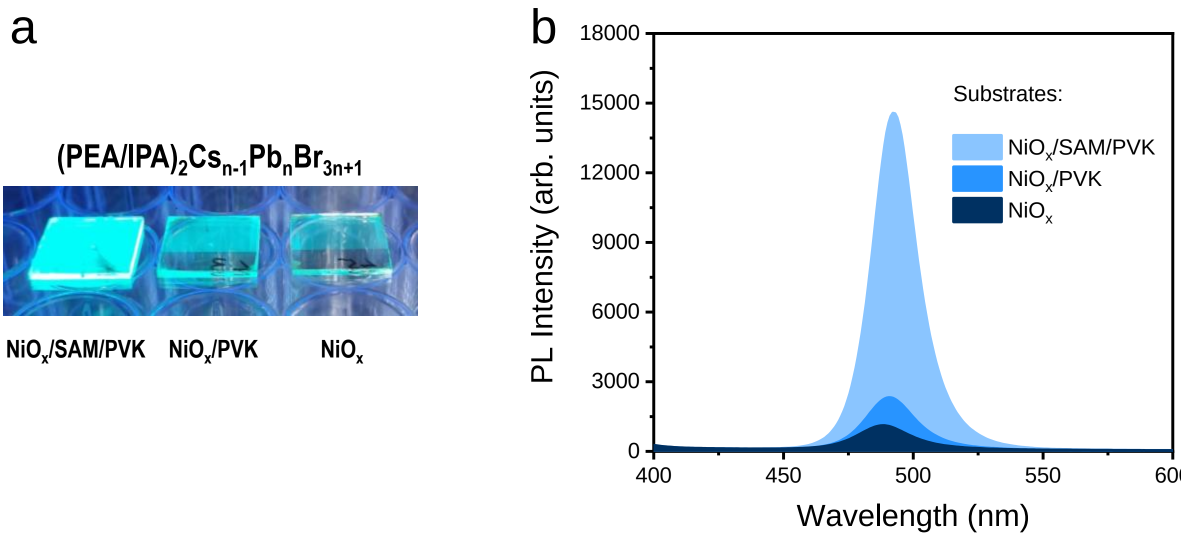

Supplementary Figure 8 | PL properties of $(PEA/IPA)_2Cs_{n-1}Pb_nBr_{3n+1}$ perovskite films on NiO_x, NiO_x/PVK and NiO_x/SAM/PVK substrates. a Photos of perovskite films under 365-nm ultraviolet lamp excitation. **b** PL spectra of the corresponding perovskite films. The slight red shift of PL from NiO_x to NiO_x/PVK to NiO_x/SAM/PVK cases is due to the increasing 3D perovskite ratio in quasi-2D perovskite films, according to the XRD results shown in Supplementary Figure 9.

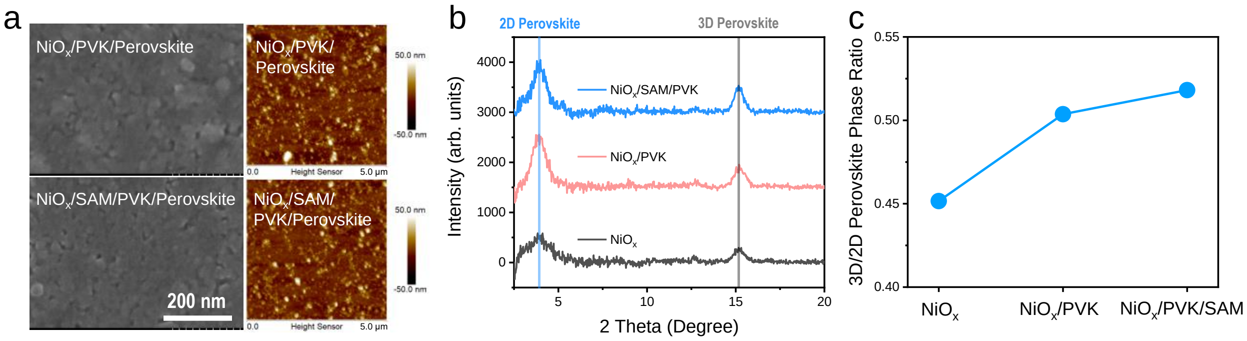

Supplementary Figure 9 | Film quality of $(PEA/IPA)_2Cs_{n-1}Pb_nBr_{3n+1}$ perovskite on various substrates. a Morphologies, Left panel: SEM images; Right panel: AFM images. In both cases, the perovskite film morphologies are similar, suggesting that introducing the 2PACz SAM had a negligible impact on the perovskite morphology. **b** X-ray diffraction patterns. The similar X-ray diffraction peak positions in all cases suggest that different substrates had a negligible impact on forming the quasi-2D perovskite phase. However, the perovskite formed at NiO_x substrate had a poorer crystallinity. **c** The intensity ratio between the 3D and 2D perovskite peaks shown in **b**. An increasing 3D perovskite phase is found when changing the substrates from NiO_x to NiO_x/PVK to NiO_x/SAM/PVK.

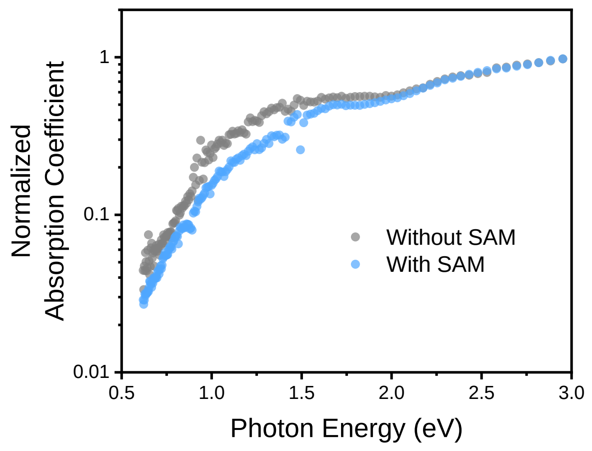

Supplementary Figure 10 | Normalized absorption coefficient (measured by photothermal deflection spectroscopy) of NiO_x film with and without SAM modification. The reduced absorption coefficient in the relatively low photo energy area in the NiO_x/SAM film indicates the reduced sub-bandgap states of NiO_x, which suggests that the SAM had successfully passivated the NiO_x surface trap states.

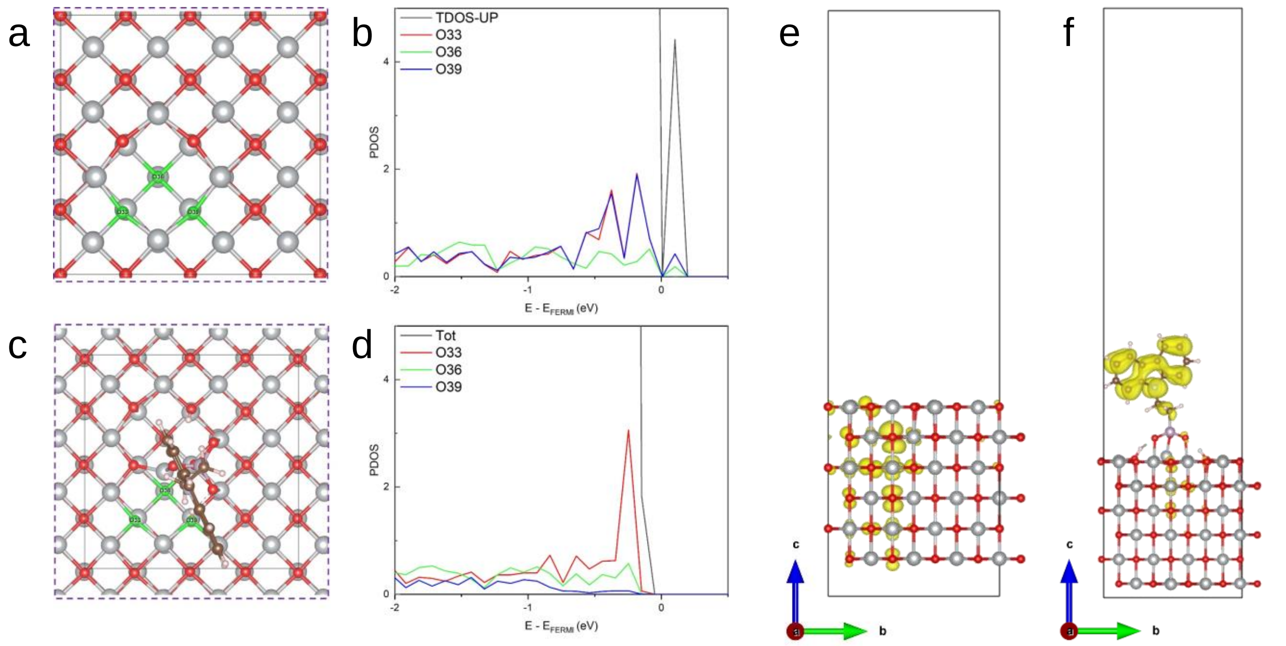

Supplementary Figure 11 | Change in NiO_x VBM after SAM modification. a Top view of the unpassivated NiO_x surface with a Ni vacancy and (**b**) corresponding spin-up PDOS, including three oxygen atoms around the Ni vacancy. **c** Top view of the passivated NiO_x surface with SAM and (**d**) corresponding spin-up PDOS, including three oxygen atoms around the Ni vacancy. The isosurface plots of VBM of the unpassivated NiO_x surface (**e**) and the passivated NiO_x surface (**f**).

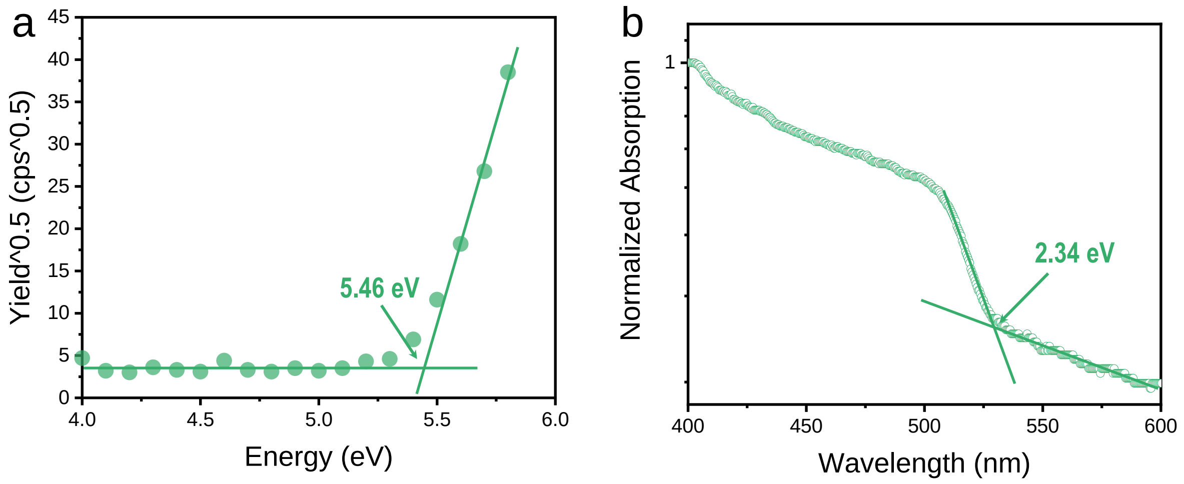

Supplementary Figure 12 | Energy level confirmation of $PEA_2(Cs_{0.933}FA_{0.067})_{n-1}Pb_nBr_{3n+1}$. a Atmospheric ultraviolet photoelectron spectroscopy spectra of perovskite, indicating its VBM of −5.46 eV. **b** Normalized absorption of perovskite, indicating its bandgap of 2.34 eV. Combining with its VBM, we calculated its conduction band minimum to be −3.12 eV.

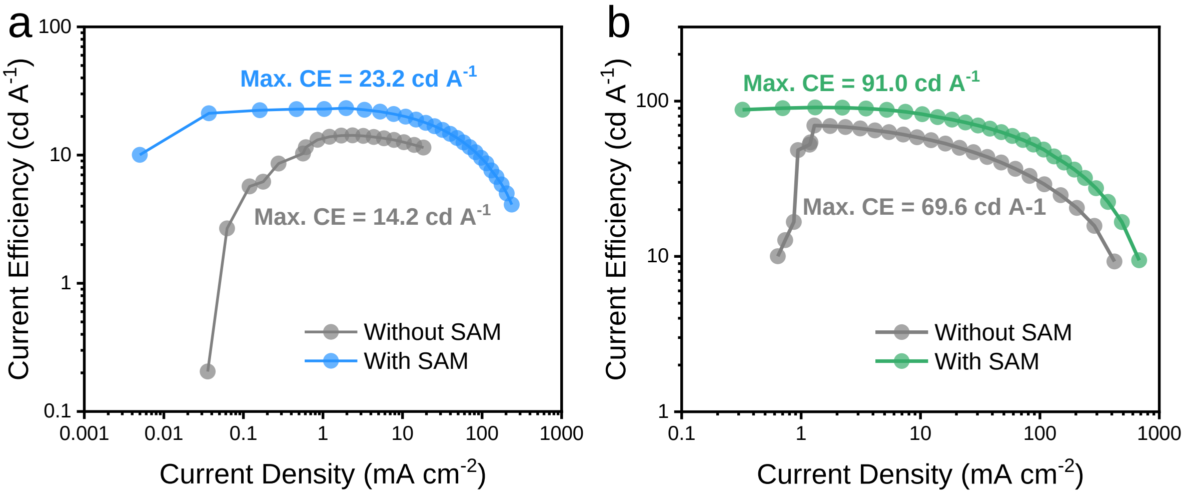

Supplementary Figure 13 | Current density–current efficiency curves of the best (a) blue and (b) green PeLEDs with and without SAM.

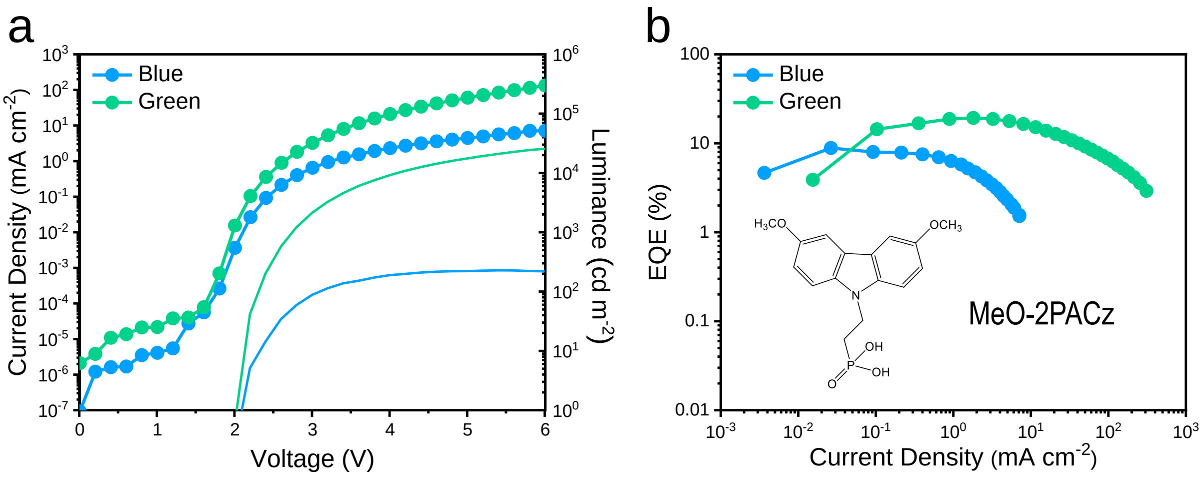

Supplementary Figure 14 | Device performance of the blue and green PeLEDs with device architecture of ITO/NiO$_x$/MeO-2PACz/PVK/Perovskite/TPBi/LiF/Al. a Current density–voltage curves (dotted lines) and luminance–voltage curves (solid lines) of the blue and green PeLEDs with MeO-2PACz layer. **b** Current density–EQE curves of the blue and green PeLEDs with MeO-2PACz layer. The best EQEs for blue and green PeLEDs are 8.8% and 19.3%, respectively. Inset is the chemical structure of the MeO-2PACz molecule.

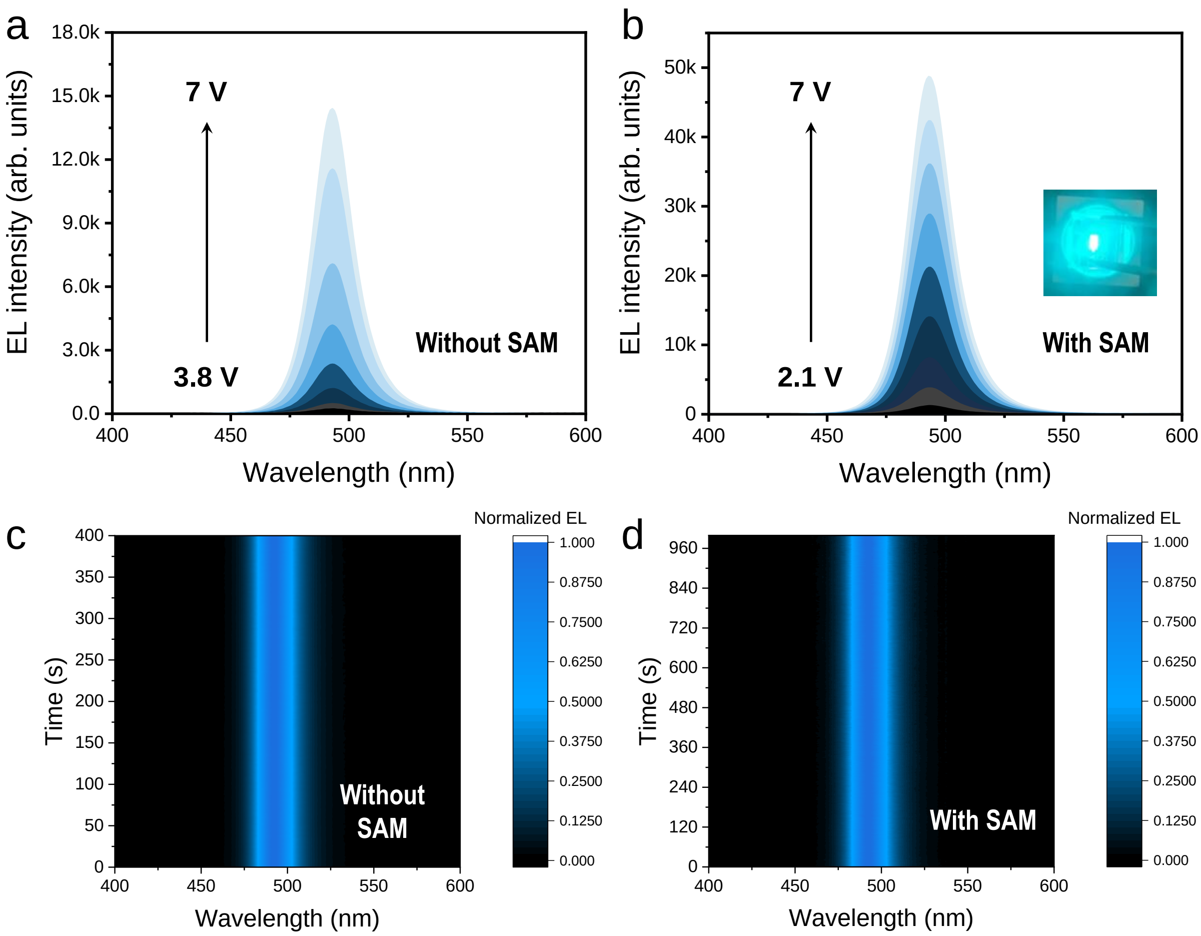

Supplementary Figure 15 | Spectrum stability of blue PeLEDs with an emission peak at ~493 nm. **a** EL spectra of the device without SAM under an applied voltage ranging from 3.8 V to 7 V. **b** EL spectra of the device with SAM under an applied voltage ranging from 2.1 V to 7 V. Inset is the photo of a working blue PeLED. Normalized EL spectra of the device (**c**) without SAM and (**d**) with SAM under continuous operation (at the max EQE). No spectrum shift was observed in any case.

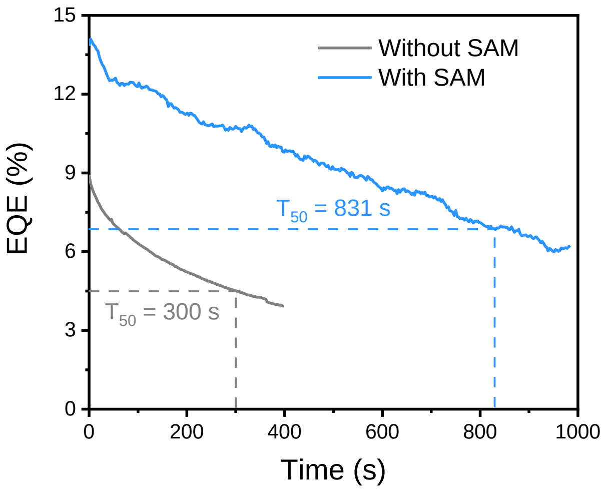

Supplementary Figure 16 | Device lifetime of the best blue PeLEDs with and without SAM. Besides the effect of the organic/inorganic heterointerfaces mentioned in the main text, electric-field-induced ion migration and phase segregation, electrical stress, and Joule heat are also considered the factors that lead to the degradation of our devices[4–9].

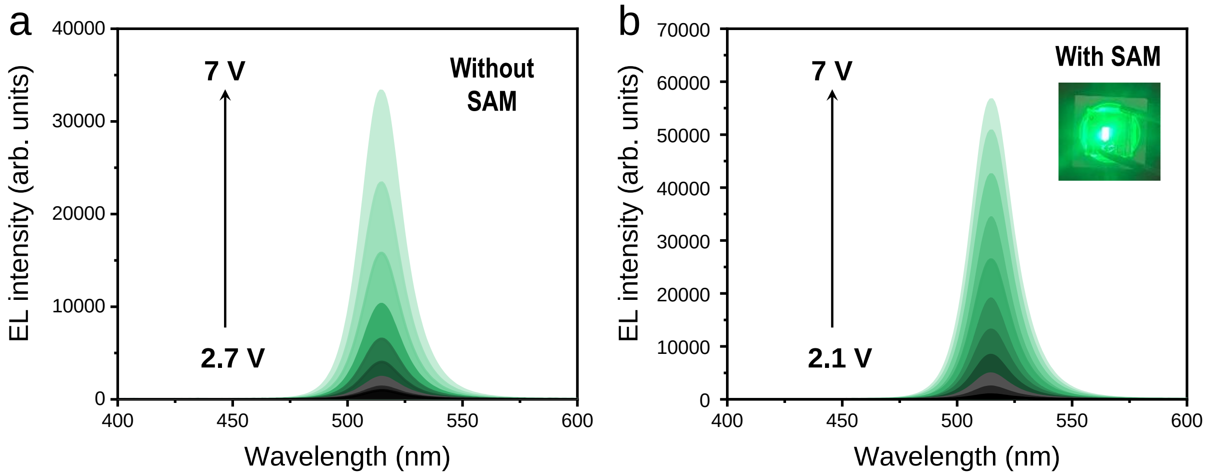

Supplementary Figure 17 | Spectrum stability of green PeLEDs with an emission peak at ~515 nm. a EL spectra of the device without SAM under an applied voltage ranging from 2.7 V to 7 V. **b** EL spectra of the device with SAM under an applied voltage ranging from 2.1 V to 7 V. Inset is the photo of a working green PeLED.

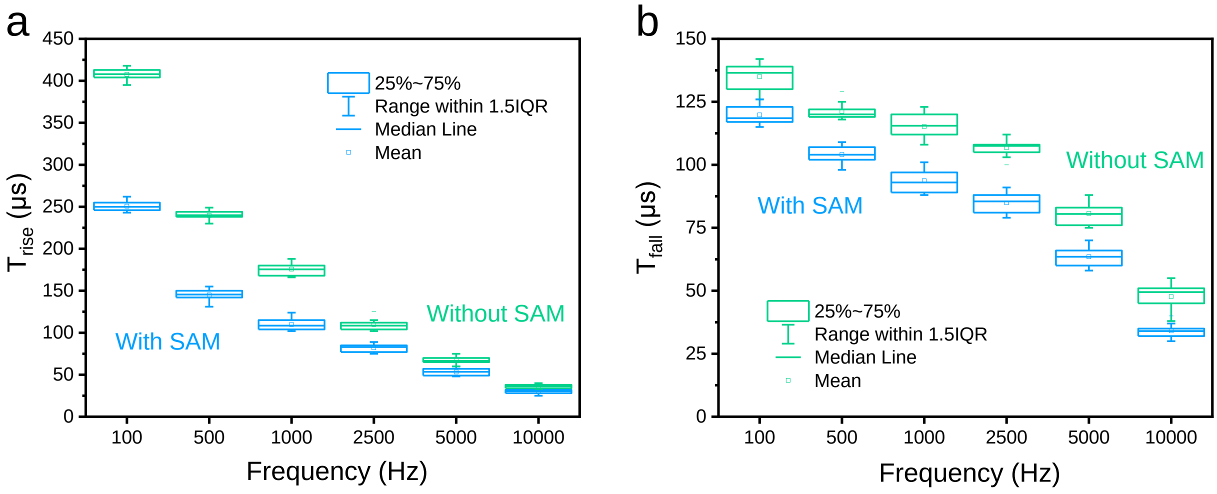

Supplementary Figure 18 | Statistical data of (a) T_{rise} and (b) T_{fall} under 100, 500, 1000, 2500, 5000 and 10000 Hz of the PeLEDs with and without SAM modification. The IQR represents interquartile range.

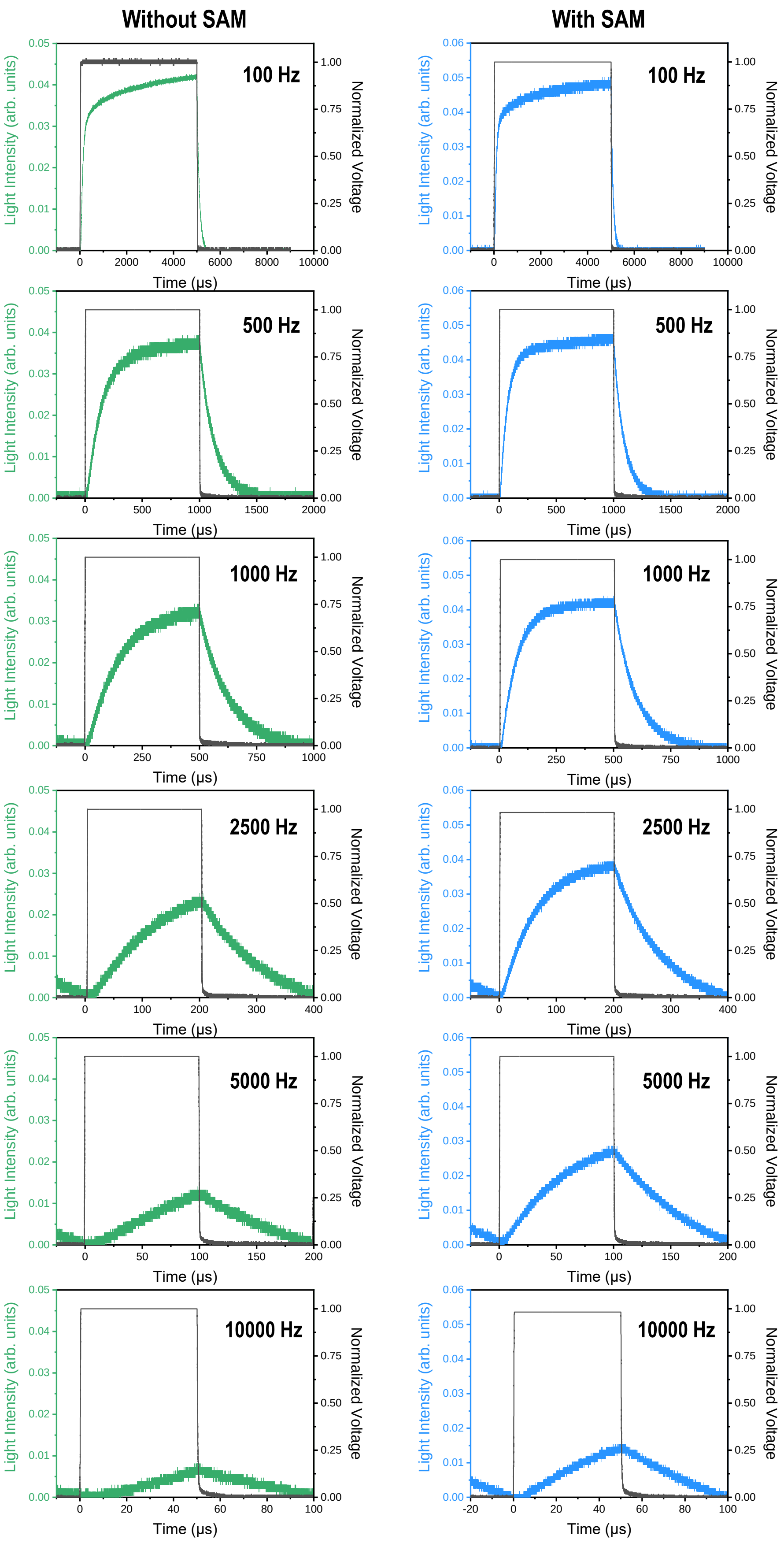

Supplementary Figure 19 | On-off response of PeLEDs with and without SAM at different frequencies. The applied voltage was 7 V.

Supplementary Table

Supplementary Table 1 | Summary of stability of the blue PeLEDs in the past 4 years.

Perovskite composition	EL peak (nm)	Testing mode	Testing condition	Lifetime	Ref.
$CsPbBr_3$ QDs	480	Constant current density	L_0=100 cd m^{-2}	126 min	[10]
$(PCTA)_2CsPb_2Br_7$	480	Constant current density	L_0=67 cd m^{-2} J_0=1 mA cm^{-2}	21.6 min	[11]
$CsPbBr_{3-x}Cl_x$ + CsFA-Ac	477	Constant current density	L_0=100 cd m^{-2}	120 s	[12]
$PEA_2(Rb_xCs_{1-x})_{n-1}Pb_n(Br_{1-y}Cl_y)_{3n+1}$	475	Constant bias	L_0=100 cd m^{-2} V_0=3.5 V	100 s	[13]
$CsPbBr_3$ QDs	469	Constant current density	L_0=115 cd m^{-2} J_0=12.5 mA cm^{-2}	25 h	[14]
$PEA_2(Cs_{1-x}EA_xPbBr_3)_2PbBr_4$	488	Constant current density	L_0=100 cd m^{-2} J_0=1.5 mA cm^{-2}	1h	[15]
$PEA_2Cs_{n-1}Pb_n(Cl_xBr_{1-x})_{3n+1}$	480	Constant bias	V_0=4.4 V	10 min	[16]
PEACl + $CsPbBr_3$ + YCl_3	485	Constant bias	L_0=100 cd m^{-2} V_0=3.2 V	80 min	[17]
$PEA_2Cs_{2-x}EA_xPb_3Br_{10}$	490	Constant current density	L_0=60 cd m^{-2} J_0=0.44 mA cm^{-2}	55.3 min	[18]
$(Cs/Rb/K/PEA)Pb(Br/Cl)_3$	488	Constant current density	L_0=100 cd m^{-2}	5.12 min	[19]
$CsPb(Cl/Br)_3$	487	Constant current density	L_0=178 cd m^{-2} J_0=1 mA cm^{-2}	2900 s	[20]
p-F-$PEA_2Cs_{n-1}Pb_n(Cl_xBr_{1-x})_{3n+1}$	489	Constant current density	J_0=1 mA cm^{-2}	10.4 min	[21]
$(PEA/IPA)_2Cs_{n-1}Pb_nBr_{3n+1}$	493	Constant current density	L_0=100 cd m^{-2} J_0=1 mA cm^{-2}	~14 min	This work

Supplementary References

1. Alghamdi, A. R. M., Yanagida, M., Shirai, Y., Andersson, G. G. & Miyano, K. Surface passivation of sputtered NiO_x using a SAM interface layer to enhance the performance of perovskite solar cells. *ACS Omega* **7**, 12147–12157 (2022).

2. Cheng, M., Fan, H., Song, Y., Cui, Y. & Wang, R. Interconnected hierarchical $NiCo_2O_4$ microspheres as high-performance electrode materials for supercapacitors. *Dalton Trans.* **46**, 9201–9209 (2017).

3. Sun, J. et al. NiO_x-seeded self-assembled monolayers as highly hole-selective passivating contacts for efficient inverted perovskite solar cells. *Sol. RRL* **5**, 2100663 (2021).

4. Xing, J. et al. Color-stable highly luminescent sky-blue perovskite light-emitting diodes. *Nat. Commun.* **9***, 3541 (2018).*

5. Carrillo, J. et al. Ionic reactivity at contacts and aging of methylammonium lead triiodide perovskite

solar cells. *Adv. Energy Mater.* **6**, 1502246 (2016).

6. Chen, S. et al. Mobile ion induced slow carrier dynamics in organic–inorganic perovskite $CH_3NH_3PbBr_3$. *ACS Appl. Mater. Interfaces* **8**, 5351–5357 (2016).

7. Ahn, N. et al. Trapped charge-driven degradation of perovskite solar cells. *Nat. Commun.* **7**, 13422 (2016).

8. Tyagi, P., Srivastava, R., Indu Giri, L., Tuli, S. & Lee C. Degradation of organic light emitting diode: heat related issues and solutions. *Synthetic Metals* **216**, 40–50 (2016).

9. Zou, G. et al. Color-stable deep-blue perovskite light-emitting diodes based on organotrichlorosilane post-treatment. *Adv. Funct. Mater.* **31**, 2103219 (2021).

10. Jiang, Y. et al. Synthesis-on-substrate of quantum dot solids. *Nature* **612**, 679–684 (2022).

11. Zhou, Y.-H. et al. Spectral stable blue perovskite light-emitting diodes by introducing organometallic ligand. *Adv. Opt. Mater.* **10**, 2101655 (2022).

12. Ding, W. et al. Transformation of quasi-2D perovskite into 3D perovskite using formamidine acetate additive for efficient blue light-emitting diodes. *Adv. Funct. Mater.* **32**, 2105164 (2022).

13. Yang, Y. et al. Highly efficient pure-blue light-emitting diodes based on rubidium and chlorine alloyed metal halide perovskite. *Adv. Mater.* **33**, 2100783 (2021).

14. Bi, C. et al. Suppressing auger recombination of perovskite quantum dots for efficient pure-blue light-emitting diodes. *ACS Energy Lett.* **8**, 731–739 (2023).

15. Chu, Z. et al. Large cation ethylammonium incorporated perovskite for efficient and spectra stable blue light-emitting diodes. *Nat. Commun.* **11**, 4165 (2020).

16. Li, Z. et al. Modulation of recombination zone position for quasi-two-dimensional blue perovskite light-emitting diodes with efficiency exceeding 5%. *Nat. Commun.* **10**, 1027 (2019).

17. Wang, Q. et al. Efficient sky-blue perovskite light-emitting diodes via photoluminescence enhancement. *Nat. Commun.* **10**, 5633 (2019).

18. Liu, S. et al. Zwitterions narrow distribution of perovskite quantum wells for blue light-emitting diodes with efficiency exceeding 15%. *Adv. Mater.* **35**, 2208078 (2022).

19. Zhu, C. et al. High triplet energy level molecule enables highly efficient sky-blue perovskite light-emitting diodes. *J. Phys. Chem. Lett.* **12**, 11723–11729 (2021).

20. Shen, Y. et al. Multifunctional crystal regulation enables efficient and stable sky-blue perovskite light-emitting diodes. *Adv. Funct. Mater.* **32**, 2206574 (2022).

21. Xia, Y. et al. Reduced confinement effect by isocyanate passivation for efficient sky-blue perovskite light-emitting diodes. *Adv. Funct. Mater.* **32**, 2208538 (2022).
